# Viral Load, Integration and Methylation of E2BS3 and 4 in Human Papilloma Virus (HPV) 16-Positive Vaginal and Vulvar Carcinomas

**DOI:** 10.1371/journal.pone.0112839

**Published:** 2014-11-13

**Authors:** Gabriella Lillsunde Larsson, Gisela Helenius, Bengt Sorbe, Mats G. Karlsson

**Affiliations:** 1 Department of Laboratory Medicine, Örebro University Hospital, Örebro, Sweden; 2 School of Health and Medical Sciences, Örebro University, Örebro, Sweden; 3 Department of Oncology, Örebro University Hospital, Örebro, Sweden; Institut national de la santé et de la recherche médicale, France

## Abstract

**Objective:**

To investigate if viral load, integration and methylation of E2BS3 and 4 represent different ways of tumor transformation in vaginal and vulvar carcinoma and to elucidate its clinical impact.

**Methods:**

Fifty-seven samples, positive for HPV16, were selected for the study. Detection of viral load was made with realtime-PCR using copy numbers of E6 and integration was calculated from comparing E2 to E6-copies. Methylation of E2BS3 and 4 was analysed using bisulphite treatment of tumor DNA, followed by PCR and pyrosequencing.

**Results:**

Vaginal tumors were found to have a higher viral load (p = 0.024) compared to vulvar tumors but a high copy number (> median value, 15 000) as well as high methylation (>50%) was significantly (p = 0.010 and p = 0.045) associated with a worse cancer-specific survival rate in vulvar carcinoma, but not in vaginal carcinoma. Four groups could be defined for the complete series using a Cluster Two step analysis; (1) tumors holding episomal viral DNA, viral load below 150 000 copies not highly methylated (n = 25, 46.3%); (2) tumors harboring episomal viral DNA and being highly methylated (>50%; n = 6, 11.1%); (3) tumors with viral DNA fully integrated (n = 11, 20.4%), and (4) tumors harboring episomal viral DNA and being medium- or unmethylated (<50%) and having a high viral load (> total mean value 150 000; n = 12, 22.2%). The completely integrated tumors were found to be distinct group, whilst some overlap between the groups with high methylation and high viral load was observed.

**Conclusion:**

HPV16- related integration, methylation in E2BS3 and 4 and viral load may represent different viral characteristics driving vaginal and vulvar carcinogenesis. HPV16- related parameters were found to be of clinical importance in the vulvar series only.

## Introduction

Vaginal and vulvar carcinomas are small groups among the gynecological malignancies, together constituting about 5% of all female genital cancers in Sweden [1]. In both tumor types the human papilloma virus (HPV) can be detected and the viral prevalence differs between tumor sites, with HPV-positive tumors being more common in vaginal compared with vulvar carcinomas. A majority of the HPV-positive cases are reported to be positive for the genotype 16 [2]–[5].

HPV16 has been extensively studied in cervical carcinoma but the detailed knowledge on this genotype is far less in vaginal and vulvar carcinomas. HPV16 is known to be the most carcinogenic genotype in cervical cancer development, due to its propensity to become persistent and to its ability to induce changes in the cell cycle regulation [6]. Specific variants of HPV16 have also been associated with difference in persistence and cancer development [7], [8]. High levels of the viral oncoproteins E6 and E7 in proliferating cells that are not shed by the epithelia are a necessity for oncogenic transformation and can be established if the virus integrates its DNA into the host cell chromosomes [9], [10]. The viral expression is controlled by the viral long control region (LCR), where both viral and host cell factors bind and affect transcription [11], [12]. Upon integration, the E2 open reading frame (ORF) is known to be disrupted [13]–[17]. E2 protein is, among other things, the normal regulator of E6 and E7 expression through its binding to conserved E2- binding sites (E2BS) in the LCR [11]. The underlying event for integration is unknown [10], but when the viral genome is integrated and no E2 protein is present, high amount of E6 and E7 can be produced and lead to malignant transformation [12]. This is thought to be a key step in cervical carcinogenesis supported by in vitro studies and also by the fact that many cervical carcinoma cellines as well as clinical cancers have been found to be integrated [18]–[21]. However, recent studies have shown that precursor lesions such as CIN 1 can harbour integrated viral genome [22], [23] and that there are also carcinomas with only episomal viral DNA present [15], [24]–[26].

An alternative mechanism for the unregulated expression of oncoproteins is proposed that includes methylation of the E2- binding sites in the viral LCR. Epigenetic regulation of viral gene expression seems to be important, since methylation of HPV DNA has been shown to change according to viral life cycle but also in relation to neoplastic progression [27]–[29]. High level of methylation of the HPV-genome in relation to neoplastic progression has been shown for both structural genes [30] as for the LCR. However, for the LCR there are conflicting results showing both high and low methylation for this region [31]–[33]. Each E2BS-site contains CpG dinucleotides, nucleotides that are potential targets for methylation. There are four different E2- binding sites within the LCR. When E2-concentration is high, E2 has been shown to bind to the E2- binding sites closest to the early promoter (E2BS2, 3 and 4) thereby blocking transcription from the early promoter [11], [12], [34]–[36]. Hence, methylation of the ones close to the early promoter could activate transcription and it has also been shown *in vitro* that methylation of the CpG dinucleotides inhibits the binding of E2 [37]. Also, further evidence comes from a study by Fernandez et al where E6/E7 gene expression from the highly methylated cancer celline CaSki is reduced when treated with a demethylating agent [38].

Viral load is also suggested to be a key player in lesion development, by sustaining high amounts of E6 and E7. Many studies have tried to evaluate viral load as a predictor of disease progression and high initial viral load has in some studies been linked to an increased risk of cytological abnormalities [39]–[41], and in some studies not [42]–[44]. Investigation of viral load in cancer has shown that tumors have varying amounts of viral DNA [45], [46] possibly reflecting the viral status of integration.

Viral integration and methylation of E2BS3 and 4 could therefor represent two different mechanisms to interfere with E2 function, increase oncogene expression and potentially lead to transformation. Studies on cervical samples have also shown that integrated viral genomes are less likely to be highly methylated [25], [33], [47], [48]. High viral load may represent a third scenario in tumors that are not driven by integration or E2BS- methylation strategies.

We have in previous studies on vaginal and vulvar carcinomas found the distribution of HPV16- variants to vary between the series. Also, for vulvar carcinoma there was a trend towards worse survival for variant group E–G131, a variant group that was not as abundant in the vaginal series. This leads us to speculate that differences might be present between the series in regards of viral status, methylation of E2BS3 and 4 and viral load that could affect tumor genesis and patient outcome. Also, since previous studies on integration and methylation of E2BS3 and 4 has included cervical samples only, this study could shed light on whether vaginal and vulvar carcinomas has equal levels of viral load, and same amount of integration and methylation of E2BS3 and 4.

The aim of this study is therefore to investigate viral load, integration and viral methylation of E2BS3 and 4 in two series of HPV16-positive vaginal and vulvar carcinomas. Furthermore, the viral characteristics are also analysed in relation to the HPV16- variants present in both series.

## Materials and Methods

### Material

Fifty-seven samples, positive for HPV16, from two consecutive series of 69 vaginal carcinomas and 133 vulvar squamous cell carcinoma were selected for the study. Thirty-one of the vulvar carcinomas and 26 of the vaginal carcinomas were further analysed for viral load, integration and methylation status of E2BS3 and 4. Samples were collected between 1972 and 2008. Clinical data were retrieved from patient records at the Department of Oncology, Örebro University Hospital.

### Ethics statement

The study was approved by the regional Ethical Committee in Uppsala, Sweden (EPN, Dnr 2008/294). Patients were orally informed about the clinical research database and after 2003 also about tissue biobanking according to the Swedish biobank act 2002∶297. No specific informed consent was requested by the Ethical Committee.

### DNA extraction, genotyping and variant testing of HPV16

All tumor samples were formalin fixed and paraffin embedded. Paraffin blocks were cut in 4 µM sections and tumor area was marked on hematoxylin and eosin stained sections (M.K). Tissue cores were selected from tumor areas with high tumor cell content.

DNA was extracted from formalin fixed and paraffin embedded samples (FFPE) and genotyped as previously described. For samples positive for HPV16, variant of HPV16 was investigated [3], [4] and the same DNA extraction was used for the following analyses of viral load, integration and methylation of E2BS3 and 4.

### Viral load and integration of HPV16

Detection of the E2 and E6 gene for HPV16 was performed with realtime-PCR on the 7500 Fast Real-Time PCR System (Thermo Fisher Scientific, Waltham, USA). Standard curves for the genes E2 and E6 were established by making a serial dilution of the plasmid pBR322 containing the total HPV16-genome in a background of human DNA. Dilutions were made to equal 3, 30, 300, 3000, 30 000 and 300 000 copies of HPV16 E2 and E6.

Samples were analysed in triplicates using 20 µl reactions containing 1× Taqman Fast Universal PCR Mastermix and 0.3 µM forward (E2: aac gaa gta tcc tct cct gaa att att ag, E6: gag aac tgc aat gtt tca gga cc and reverse primer (E2: cca agg cga cgg ctt tg [46]), E6: tgt ata gtt gtt tgc agc tct gtc c) together with 0.1 µM probe (E2: Fam-cac tcc gcc gcg acc cat a BHQ, E6: Fam-cag gag cga ccc aga aag tta cca cag tt-BHQ [46] (Thermo Fisher Scientific). After an initial denaturation at 95°C for 20 seconds, reaction mixtures underwent 45 cycles at 95°C for 3 seconds followed by 60°C for 30 seconds. Results were analysed by software 7500 Fast System SDS Software (Thermo Fisher Scientific).

Viral load was estimated as copy numbers of E6 in 20 ng of DNA. Integration status was calculated by dividing copy numbers of E2 to E6 where ratios of 1 and above indicated an excess of episomal genome. Ratios below 1 equalled a mixed genome and fully integrated genome lacked the E2 gene completely.

### Methylation of E2BS3 and 4

Samples were treated with bisulphite using EZ DNA Methylation-Gold according to manufacturer’s instructions (Zymo research, Irvine, USA). PCR for bisulphite treated DNA of the region covering nucleotide positions 37, 43, 52 and 58, (reference sequence NC_001526 ) was performed using 0.5 µM forward primer (biotin-tta taa taa ttt atg tat aaa att aag gg) and 0.5 µM reverse primer (aat tct ctt tta ata cat aaa ata tct act) (Sigma Aldrich, St. Louis, USA). Design of amplicon was made using Pyromark Assay design (CpG) (Qiagen, Hilden, Germany). Reaction mixtures included 1× EpiTect Master Mix (Qiagen), 0.25 mM MgCl (Thermo Fisher Scientific) and 5 µl of bisulphite treated DNA. After an initial denaturation at 95°C for ten minutes, reaction mixes underwent 45 cycles of 94°C for 15 seconds, 42°C for 30 seconds, 72°C for 30 seconds followed by 72°C for ten minutes on PCR equipment EppendorfMastercycler ep gradient S (Eppendorf AG, Hamburg, Germany). Amplicons were verified by size on agarose gel before analysed with pyrosequencing.

Samples were prepared for pyrosequencing using the Vacuum prep Workstation (Qiagen). Single-strand sequencing template was transferred to a 96-well sequencing plate containing 1 µM sequencing primer (aaa ata tct act ttt ata cta)(Sigma Aldrich). The plate was incubated in 80°C for two minutes. Sequencing was performed in a PSQ 96 MA system using PyroMark Gold Q96 Reagents (Qiagen). The results were analysed in the Pyro Q-CpG Software. For each sample duplicate reactions were used. Results from each position (mean of both samples) were manually addressed. Methylation result from SiHa cell line was used as cut-off level for methylation-positive samples. Methylated samples were further divided into groups of medium methylated (11–50%), and highly methylated (51–100%).

### Controls

DNA from cell lines SiHa (ATCC HTB-35) and CaSKi (ATCC CRL-1550) were used as positive controls. SiHa cells are known to harbour 1 to 2 copies of integrated HPV16 with a deletion of the E2 open reading frame (ORF) [49] and lacking methylation in E2BS3 and 4 [50]. CaSKi cells carry about 600 copies of integrated HPV16 [51], with the E2 ORF intact [49]. CaSKi E2BS3 and 4 are highly methylated [50]. The human cell line HEL (ATCC TIB-180) was used as negative control.

### Statistics

For viral load evaluation between cohorts medians were used due to the distribution of data (skewed). A cut-off level of 150 000 copies was used for defining an extreme group of high viral load and was selected from the mean value of viral load from both series combined.

For analysis of integration; tumors with mixed and episomal DNA were grouped together and compared with fully integrated tumors. For analysis of methylation; a 50% cut-off level for high methylation was used, calculated from the average for the four positions of 37, 43, 52 and 58. A Cluster Two step analysis was performed on all samples for grouping analysis using SPSS Statistics (version 22, IBM, New York, USA).

Differences in viral load between groups were tested by Mann-Whitney U-test. For comparison of proportions we used the Pearson chi-square test. The Kaplan-Meier technique was used for generating survival curves and the log-rank test was used to test for differences. Cox proportional multivariate regression analysis with cancer-specific survival as the outcome variable was used for multivariate modeling.

All p values were based on two-sided tests, with *p*<0.05 considered statistically significant. The Statistica software packages (version 12, StatSoft, Inc., Tulsa, USA) as well as SPSS Statistics (version 22, IBM, New York, USA) were used for the statistical analyses.

## Results

### Vaginal and Vulvar Cohorts

In order to investigate the biological differences between the cohorts, both series were investigated for viral load, integration and methylation of E2BS3 and 4.

#### Viral load

Viral load in the vaginal cohort varied between 284 and 667 417 E6 copies with a median value of 91 721 copies (lower quartile 14 242, upper quartile 325 054) and in the vulvar cohort the viral load varied between 499 and 1 477 442 copies of E6 with a median value of 14 676 copies (lower quartile 3410, upper quartile 84 582).

**Figure 1:**
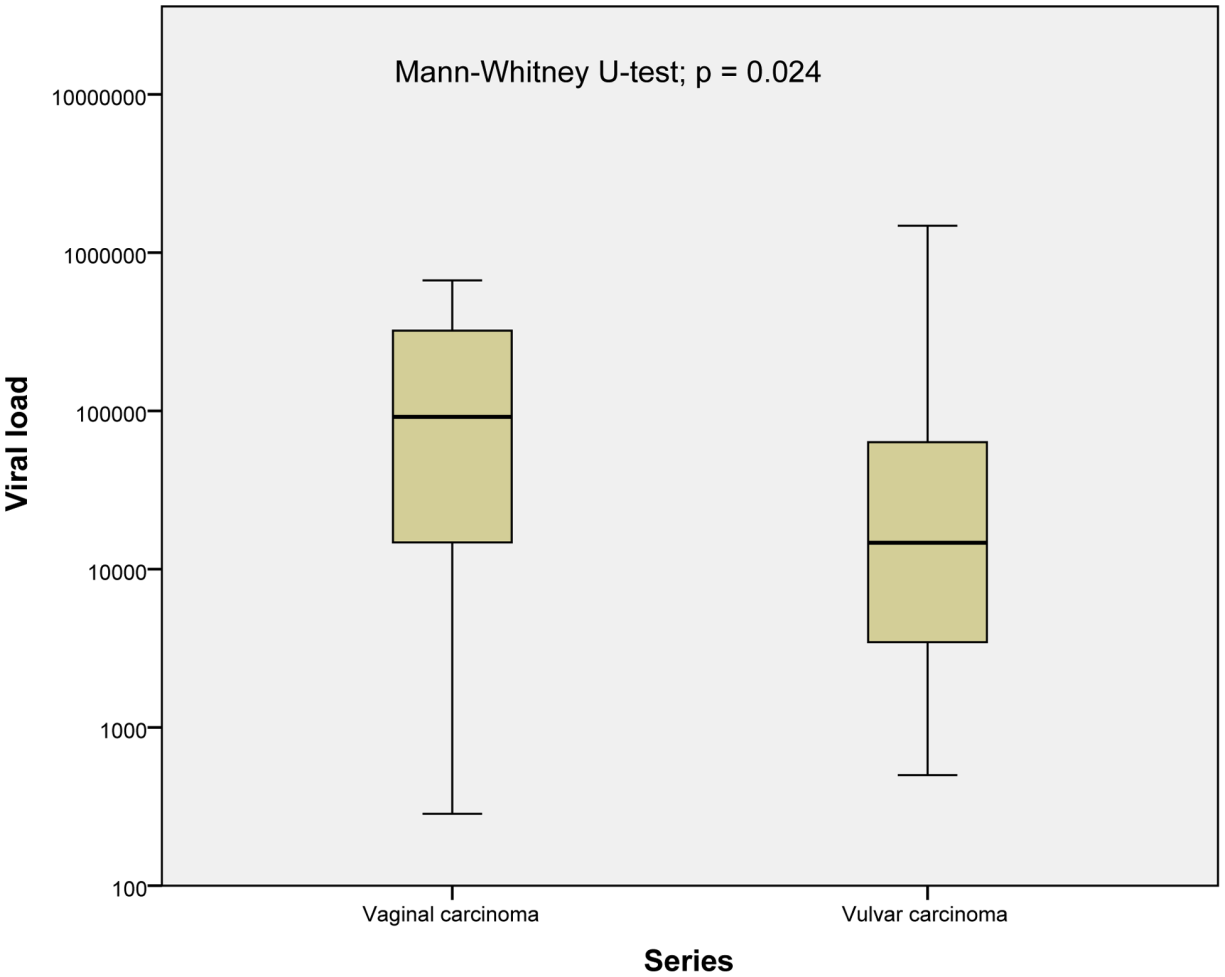
Box plot showing differences in HPV16 viral load between vaginal and vulvar series. The difference in viral load between vaginal and vulvar carcinomas was significant (Mann-Whitney U-test; p = 0.024).

The vaginal series harboured more tumors with high viral load resulting in a higher median value, and there was a statistically significant difference between viral load in the two series (Mann-Whitney U-test; p = 0.024, Figure 1).

#### Integration

In both series, few tumors were integrated. In the vaginal cohort, 15.4% had complete (4/26) integrated viral genome, 15.4% had a mix (4/26) of integrated and episomal viral DNA and 69.2% (18/26) carried the virus in the pure episomal state. For the vulvar cohort, 22.6% (7/31) had total integrated viral genome, 19.4% (6/31) had a mix of the two and 58.0% (18/31) carried the virus in the pure episomal state.

#### Methylation of E2BS3 and 4

When comparing levels of methylation, in the vaginal series 15.4% (4/26) of the tumors were highly methylated (>50%), 11.5% (3/26) were medium methylated and 73.1% (19/26) were unmethylated. The vulvar cohort had 7.1% (2/28) highly methylated tumors, 7.1% (2/28) medium methylated tumors and 85.8% (24/28) that were unmethylated. Three of the vulvar cases were excluded due to insufficient material or to inconclusive PCR results. Methylation levels in sites 37, 43, 52 and 58 ranged from 11% to 96%, table 1.

**Table 1:** Distribution of methylation in percentages (%) for each position in E2BS3 and 4 in the two series.

Nucleotide positions, ref seq NC_001526						
Sample no	Series	E2BS3		E2BS4		Mean value
(n = 54)	Vaginal or vulvar	37	43	52	58	all positions
2	Vaginal	76	79	79	70	76
4	Vaginal	75	78	79	76	77
7	Vaginal	76	79	82	75	78
8	Vaginal	17	18	18	18	18
18	Vaginal	13	15	16	20	16
20	Vaginal	72	76	74	66	72
23	Vaginal	30	33	32	28	31
6	Vulvar	11	12	12	12	12
10	Vulvar	90	95	96	91	93
20	Vulvar	71	80	80	74	76
24	Vulvar	42	40	45	40	42

In total series, 6 samples, of 54 samples analysed, showed high methylation (>50%) and 5 samples fulfilled the criteria of medium methylation.

The vaginal series had numerically more tumors that were highly methylated compared with the vulvar series that instead had more tumors that were fully integrated, but neither of these findings were significant (Pearson chi-square; p = 0.493 and p = 0.336 ).

#### Clinical outcome

**Figure 2:**
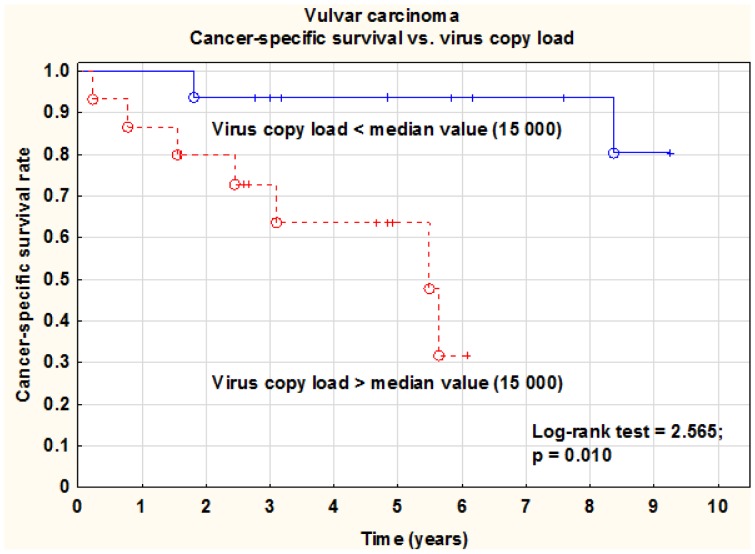
Cancer-specific survival rate versus HPV virus copy load in vulvar carcinomas. There was a statistically significant (log-rank test; p = 0.010) difference in cancer-specific survival rate between patients with tumors showing low (< median value) and high (> median value) virus loads. Median value of viral load for vulvar series was used (15 000).

To further investigate the biological differences between the cohorts, with regard to patient outcome, both series were investigated for cancer-specific survival in relation to viral load, integration and methylation of E2BS3 and 4. Despite the fact that the median number of virus copies was higher for the vaginal than for the vulvar series, a high copy number (> median value, 15 000) was highly significantly (log-rank test; p = 0.010) associated with a worse cancer-specific survival rate in vulvar carcinoma (Figure 2), but not in vaginal carcinoma (> median value 91 000, log-rank test; p = 0.551). Integration could not be significantly associated with cancer-specific survival rate in any of the two series, log-rank test; p = 0.729, p = 0.567. High methylation (>50%) was statistically significantly associated with a worse cancer-specific survival rate in vulvar carcinoma (log-rank test; p = 0.045), but not in vaginal carcinoma (log-rank test; p = 0.122).

In a Cox proportional multivariate regression analysis including virus copy numbers, virus integration and E2BS3 and 4 methylation; with cancer-specific survival as the outcome variable, the result was different for the vulvar and the vaginal cohorts. In the vulvar series only virus copy number was statistically significant (Wald statistics; p = 0.030) and independent of the other two variables, but no significant findings were seen in the vaginal series, table 2.

**Table 2:** Cox multivariate regression analysis of integration, methylation of E2BS3 and 4 and viral load in the two series.

Variable	p-value	Risk ratio	95% CI of risk ratio	n
**Vaginal series**				
HPV-DNA integration	0.616	0.509	0.036–7.144	4
0.865				
0.220				
−3.408				
HPV-DNA methylation	0.136	4.512	0.621–32.736	4
Virus copy load >91 000	0.721	0.698	0.097–5.044	13
**Vulvar series**				
HPV-DNA integration	0.472	0.488	0.069–3.450	7
0.865				
0.220				
−3.408				
HPV-DNA methylation	0.438	1.926	0.367–10.106	2
Virus copy load >15 000	**0.030**	14.068	1.298–152.450	15

Cancer-specific survival rate is the measured endpoint. HPV-DNA integration (100%) vs. episomal or mixed episomal and integrated DNA. HPV-DNA methylation >50% vs. DNA-methylation <50%. For virus copy loads mean values of each series is used.

### Combined Viral Characteristics

To follow the hypothesis that high viral load, integration and methylation of E2BS3 and 4 are different elements leading to tumor development, four groups were defined for the complete series of vaginal plus vulvar carcinomas (n = 54) using a Cluster Two-step analysis. The groups were defined as follows: (1) tumors with episomal viral load (mixed or only episomal), viral load below 150 000 copies and not highly methylated (n = 25, 46.3%); (2) tumors harboring episomal viral DNA and being highly methylated (>50%; n = 6, 11.1%); (3) tumors with viral DNA fully integrated (n = 11, 20.4%), and (4) tumors, harboring episomal viral DNA and being medium- or unmethylated (<50%) and having a high viral load (> total mean value 150 000; n = 12, 22.2%).

**Figure 3:**
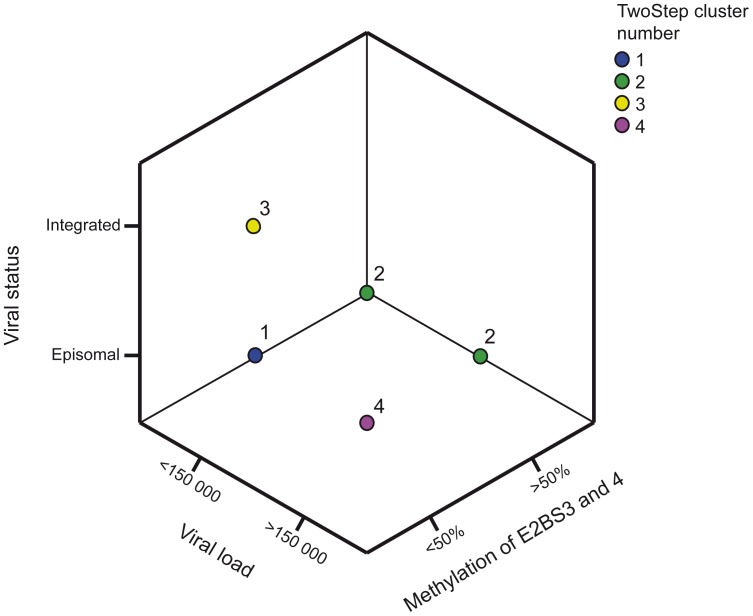
Cluster analysis data plotted in a 3-D box. Cluster identification: 1 = Mixed group (n = 25), 2 = Methylation >50% (n = 6), 3 = Integrated viral DNA (n = 11) and 4 = Viral load >150 000 (n = 12). Integrated tumors were found to be exclusive from the other groups. Overlap was seen between 4 highly methylated tumors that also had a high viral load (group 2).

The classification seemed to be distinct for tumors that were integrated since none of them were highly methylated or having a high viral load. There were however some overlap between the tumor groups with high methylation in E2BS3 and 4 and a high viral load, table 3 and Figure 3. Tumors belonging to the first group all presented a low viral load. They could further be grouped as either mixed episomal/integrated and medium methylated (n = 2) or mixed episomal/integrated and unmethylated (n = 5). Also the fully episomal tumors were either medium methylated (n = 1) or unmethylated (n = 17).

**Table 3:** Individual sample data from vaginal and vulvar tumors on viral status, viral methylation and viral load (E6 copy number) together with cluster information.

	Viral status	Methylation E2BS3 and 4	Viral load (E6)	Comment	Cluster
1	Mixed	Medium	22104		1
2	Mixed	Medium	1243		1
3	Mixed	Unmethylated	6890		1
4	Mixed	Unmethylated	36395		1
5	Mixed	Unmethylated	5209		1
6	Mixed	Unmethylated	14736		1
7	Mixed	Unmethylated	97478		1
8	Episomal	Medium	16242		1
9	Episomal	Unmethylated	99676		1
10	Episomal	Unmethylated	75048		1
11	Episomal	Unmethylated	4084		1
12	Episomal	Unmethylated	6484		1
13	Episomal	Unmethylated	4622		1
14	Episomal	Unmethylated	4151		1
15	Episomal	Unmethylated	12996		1
16	Episomal	Unmethylated	3494		1
17	Episomal	Unmethylated	10939		1
18	Episomal	Unmethylated	84582		1
19	Episomal	Unmethylated	17156		1
20	Episomal	Unmethylated	112112		1
21	Episomal	Unmethylated	47464		1
22	Episomal	Unmethylated	30634		1
23	Episomal	Unmethylated	135922		1
24	Episomal	Unmethylated	25525		1
25	Episomal	Unmethylated	26798		1
26	Episomal	High	75966		2
27	Episomal	High	85965		2
28	Episomal	High	***667417***	Overlap	2
29	Episomal	High	***1477442***	Overlap	2
30	Mixed	High	***1179742***	Overlap	2
31	Episomal	High	***243693***	Overlap	2
32	Integrated	Medium	1989		3
33	Integrated	Unmethylated	12761		3
34	Integrated	Unmethylated	30883		3
35	Integrated	Unmethylated	284		3
36	Integrated	Unmethylated	499		3
37	Integrated	Unmethylated	2941		3
38	Integrated	Unmethylated	14677		3
39	Integrated	Unmethylated	24711		3
40	Integrated	Unmethylated	2536		3
41	Integrated	Unmethylated	6576		3
42	Integrated	Unmethylated	3410		3
43	Episomal	Unmethylated	554986		4
44	Episomal	Unmethylated	321053		4
45	Episomal	Unmethylated	193802		4
46	Episomal	Unmethylated	624230		4
47	Episomal	Unmethylated	337059		4
48	Mixed	Unmethylated	212744		4
49	Episomal	Unmethylated	361842		4
50	Episomal	Unmethylated	160829		4
51	Episomal	Unmethylated	542747		4
52	Episomal	Unmethylated	535100		4
53	Episomal	Unmethylated	160178		4
54	Mixed	Medium	286019		4
55	Episomal	No result	825		
56	Episomal	No result	539		
57	Episomal	No result	2995		

High methylation refers to >50%, medium methylation between 11% to 49%.

The distribution of the above defined four groups was different, but not reaching statistical significance (Pearson chi-square; p = 0.053) between vaginal and vulvar carcinomas. The vaginal series had 4 tumors that were fully integrated (15.4%), the vulvar series 7 tumors (25.0%). High methylation was noted in 4 of the vaginal tumors (15.4%) but only in 2 tumors in the vulvar series (7.1%). Viral load >150 000 was seen in 9 tumors in the vaginal series (34.6%) and in 3 tumors in the vulvar series (10.7%). The vaginal series had 9 tumors that belonged to the mixed group (34.6%) while the vulvar series had 16 tumors in this group (57.2%).

The relation between viral status, viral load and methylation of E2BS3 and 4 was further investigated. The episomal group of tumors were found to more often have a very high viral load (16/46) compared with the fully integrated tumors (0/11; Fisher p = 0.024), but methylation was not found to be different between the designated groups (Fisher p = 0.426).

#### Clinical outcome

When analyzing for cancer-specific survival using the definition of groups within both cohorts (pooled data), no significant differences were observed between the extreme groups (2–4) and the mixed group (1; log-rank test; p = 0.112). In a Cox proportional multivariate regression analysis of the extreme groups with cancer-specific survival as the outcome variable, only high methylation was a significant and independent variable, table 4.

**Table 4:** Cox multivariate regression analysis of “extreme groups”.

Variable	p-value	Risk ratio	95% CI of risk ratio	n
HPV-DNA integration	0.836	0.865	0.220–3.408	11
0.865				
0.220				
−3.408				
HPV-DNA methylation	**0.031**	4.205	1.136–15.557	6
Virus copy load >150 000	0.525	0.658	0.181–2.394	12

Cancer-specific survival rate is the measured endpoint. HPV-DNA integration (100%) vs. episomal or mixed episomal and integrated DNA. HPV-DNA methylation >50% vs. DNA-methylation <50%. Virus copy loads >150 000 vs. copy loads <150 000 (mean value).

#### Viral load, integration and methylation of E2BS3 and 4 in the HPV16-variants

Previously reported data [3], [4] showed that the distribution of HPV16-variants differed between the series; but the dominating branches in both series were European and more specifically the variants, E-p (prototype) and E–G350, table 5.

**Table 5:** Distribution of the HPV16-variants present in both series.

HPV16-variants	Vaginal series	Vulvar series
E-p	13	13
E-G350	11	7
E-G131	1	5
E-G350+E-G131	1	2
E-C109/E-G350	–	1
E-A176	–	1
AA/NA1	–	1
No result	–	1
Total	26	31

Among the different variant groups, we found that all E–G131-variant tumors of the vulvar series had a viral load above median value (>15 000). Also, the only tumor with the variant E–G131 in the vaginal series had a high copy number (361 842), well above the median value for the vaginal series (91 721). Despite the low amount of tumors within the E–G131 group (vulvar series, n = 5), this variant group had significantly more tumors with a viral load above median value of 15 000 compared to the tumors with other HPV16-variants in this series (n = 25, Fisher’s exact test, p = 0.042). All 5 tumors from the vulvar series as well as the single tumor from the vaginal series were non-integrated as well as non- (n = 5) or medium methylated (n = 1) in E2BS3 and 4. The E–G131-variant group did not differ significantly in terms of integration or methylation of E2BS3 and 4 compared to the other variant groups.

The other two large variant groups; E-p and E–G350, did not differ significantly compared to the other variants in terms of viral load, viral integration or high methylation (>50%). The distribution of HPV16-variants within each extreme group reveled that no specific variant was more abundant than the other.

## Discussion

In this study, we have analyzed viral load, integration status and methylation of E2BS3 and 4 in a cohort of HPV16-positive vaginal and vulvar carcinomas. This is to our knowledge the first study on HPV16 characteristics such as viral load, integration and methylation of E2BS3 and 4 in vaginal and vulvar carcinomas.

In both series we found substantial variation in tumor viral load as copy numbers spanned between 281 and 1 477 442 E6-copies in the tumors analysed. Viral load estimates as a predictor of lesion development is much investigated, with conflicting results. Peitsaro et al [46], showed viral load estimates between 1 474 and 5 159 863 E6 copies/50 ng of DNA in CIN1-3 lesions. Other investigators have instead used the term normalized viral load (per cell) when estimating amount of virus in the tumor. It is possible that viral load may vary among cells in the tumor, and our estimation instead provides an average viral load based on input DNA (copy number/20 ng of input DNA). Since the extraction of tissue were made from areas where tumor cell load was classified as high, the differences seen in viral load are not likely due to variation in amount of representative cells.

In agreement with other recent studies, we found the majority of both vaginal and vulvar tumors to have the viral genome in its episomal state [15], [24], [26]. Studies on cervical carcinomas have shown that up to 50% of the tumors investigated had only episomal viral genome. In our study, 15% of the vaginal tumors and 23% of the vulvar tumors were fully integrated and 15% of the vaginal tumors and 19% of the vulvar tumors had a mixed genome (integrated and episomal viral DNA). A cell population with both integrated and episomal viral DNA will still, to some extent, produce the E2-protein that can regulate the oncogene expression. This perhaps reflects a more complex pattern of tumor genesis where a combinations of methylation of the E2BS3 and 4 and viral load plays a part. Of the mixed tumors in our cohorts, five had some level of methylation or a high viral load and five were unmethylated with a low viral load. It seems from our study that a larger amount of the vaginal and vulvar carcinomas have only episomal viral DNA compared to studies on cervical carcinomas. However, an underestimation of the amount of integrated virus genome has been shown using this method, where a tenfold excess of episomal viral genome can mask the presence of viral integrants [52].

The large majority of vaginal and vulvar tumors in our series were also non- or medium- methylated in E2BS3 and 4. Cut-off level of 50% for high methylation was set to separate tumors that were likely most under the pressure of methylation compared to other factors. Methylation was calculated from all four positions (37, 43, 52 and 58) as an average and for all tumors the difference between positions was minimal. Few studies have analyzed the E2BS3 and 4 sites in cancer samples using a quantitative method such as pyrosequencing. However, Chung et al [25] and Chaiwongkot [47] both showed varying levels of methylation in these positions using pyrosequencing for cervical cancer samples, ranging between 3% and 95%.

Despite these relatively small groups of two rare gynecological malignances, we observed some interesting tendencies between the series. One significant finding between the series was the difference in viral load. Vaginal carcinomas generally had more tumors with high E6 copy numbers, resulting in a high median value, but a high viral load for women with vulvar carcinoma, and not vaginal carcinoma, was associated with a worse prognosis. This impact of high viral load was also shown in the multivariate modeling where only virus copy number was statistically significant for the vulvar series when adjusted for integration and methylation of E2BS3 and 4. Cervical carcinomas positive for HPV16 have in several studies been shown to have a high viral load compared to pre neoplastic lesions [45], [53], [54] however viral load can also be influenced by the cell status of the virus. Also, anal carcinoma has further been shown to have high viral loads [55].

In regards of further differences between the two cohorts, there were numerically more integrated tumors in the vulvar series compared to in the vaginal series, but this observation was not significant and integration was not shown to be associated with prognosis in neither series or to be an independent variable in the multivariate analysis. There was further no significant difference in proportion of highly methylated tumors between the two series but for women with vulvar carcinoma, where high methylation of E2BS3 and 4 was seen in only two cases, this was shown to be significantly associated with a worse cancer-specific survival. However, methylation did not stand out as an independent prognostic factor in the multivariate analysis for vulvar carcinoma, where instead viral load seemed to be of most importance. Thus, our data conclude that for this series of vaginal and vulvar carcinomas, that differences in viral load and methylation of E2BS3 and 4 exist. The clinical value of integration and methylation of E2BS3 and 4 has not been extensively investigated, but Mazumder and colleagues investigated the 5-year survival for 86 patients with cervical lesions and found patients with episomal lesions to have better prognosis compared to if the viral DNA was integrated [33]. Also, a comparatively better prognosis was seen for patients with episomal methylated viral genome compared to episomal unmethylated genome. Our results are quite contradictory, and more and larger studies are needed for clinical interpretation.

To investigate the distribution of integration, methylation of E2BS3 and 4 and high viral load in tumor development, four different groups were identified spanning over both series. The groups were (1) a mixed group of tumors (n = 25), (2) highly methylated episomal tumors (n = 6), (3) integrated tumors (n = 11), and (4) high copy number episomal tumors (n = 12). One of the viral characteristics in group 2–4 could hypothetically solitary lead to increased oncogene expression and promote tumor growth and they were also clearly identified in the cluster analysis. Tumors belonging to the totally integrated group all had low virus copy number and were low or medium methylated in E2BS3 and 4. For the two other distinct groups of tumors with episomal viral DNA that were either highly methylated in E2BS3 and 4, or having a high viral copy load, there were some overlap between the groups thus, four of the tumors had both high viral load and high methylation.

When we compared the viral parameters to each other, we found the episomal tumors to more often have a high viral load, as has been described for cervical cancers [45], [48], [53] as well as for anal cancer [55]. We could not however statistically show that integrated tumors were less methylated, although only one tumor among our 11 cases had a medium methylation.

Methylation has been suggested to have parallel functions, both as a cell-antiviral mechanism to silence foreign material and as a viral strategy to favor persistence in the cell [11], [31]. Studies from several authors [25], [33], [47], [48] on cervical premalignant lesions and cervical cancer, show that episomal viral genomes were more methylated compared to integrated viral genomes. However, Chaiwongkot also showed that when multiple copies were integrated, most of them were methylated [47]. This has also been shown for the CaSki cell line that is known to harbor extensive integrated copies that are highly methylated [50], [51]. Our finding of high methylation only of episomal viral DNA follows what others have reported and indicates that when the viral genome is fully integrated, no extensive methylation of E2BS takes place. The one medium methylated case among our integrated tumors could represent a multi-copy integrant that cannot be distinguished from the others from the current data.

There were no proof of different distribution of the extreme groups between the vaginal and vulvar cohorts, however, vulvar tumors tended to be more often integrated while vaginal tumors were highly methylated and having a high viral load (p = 0.053).

Apart from the three extremes, there was also an additional group of 25 tumors (cluster 1) with more mixed properties. For this mixed group, alternative or parallel mechanisms for tumor development have to be considered. An additive combination of partial HPV integration, methylation of E2BS3 and 4 and/or moderate viral load could be in effect and induce tumor progression for tumors not belonging to the explicit groups. Different combinations of methylation of E2BS3 and 4, integration and viral load may exist where one or the other factor may influence outcome.

Alternative pathways leading to increased oncogene expression can also be considered, including mutations in the E2-binding sites, leading to loss of E2-binding properties. The four E2-binding sites have been sequenced and shown to be conserved and not subject to change [25], [47]. One further explanation for lack of repression is loss of E2-protein, despite the presence of E2-transcripts in the cell. The E2-protein is known to regulate both viral replication and the viral transcription [12] and is produced by different alternatively spliced mRNAs [56], [57]. With a new polyclonal antibody against E2, Xue et al [57] could show that a loss of E2 protein expression can in fact occur despite presence of full length transcripts. Mechanisms in transcription regulation or translation must then be in effect.

Lastly, interesting data on the HPV16-variant group E–G131 is also reported. We have in earlier publications analyzed the HPV16- variant distribution in both series of vaginal and vulvar carcinomas and its association with survival. In the vulvar series, the European variant E–G131 was then found to indicate (numerically) a worse prognosis compared to other HPV16-variants. In the present study we show that this variant group has significantly more tumors with a viral load above the median value (>15 000) compared to the other variants. This was the only significant finding when analyzing viral parameters in the different HPV 16-variant groups and this leads us to speculate that at least for vulvar carcinoma, this variant group, which holds a high viral load (>15 000), can influence patient outcome.

We conclude from this study that HPV16- related tumor groups of integration, methylation in E2BS3 and 4 and viral load could be identified in vaginal and vulvar carcinoma. Also, in vaginal and vulvar carcinomas, the fully integrated tumors carry low amounts of virus and are low methylated in E2BS3 and 4. In the clinical context, the HPV16- related parameters were found to be of importance in the vulvar series only and thus further studies on different anogenital carcinomas is therefore needed to clarify its clinical value.
